# Ph. Ricord on the Special Treatment of Phimosis and Paraphimosis

**Published:** 1840-11

**Authors:** Benjamin Dennis

**Affiliations:** Cincinnati


### Selections from American and Foreign Journals.

---

Ph. Ricord *on the special treatment of Phimosis and Paraphimosis.* Translated from the French for the Western Journal of Medicine and Surgery, by Benjamin Dennis, M. D., Cincinnati.

### OF PHIMOSIS.

Phimosis is complete or incomplete, permanent or temporary.

The permanent may be congenital or accidental, it may exist with excess in the length of the prepuce, with a prepuce not covering the entire gland, or with excess in the length of the frænum; it may have adherences to the gland, ancient or recent, complete or incomplete.

The temporary may be inflammatory or œdematous, complicated with erysipelas, with considerable tension, with gangrene, with inflammation of the gland, with blennorhagia, with chancres, with vegetations, with herpes, with perforation of the prepuce, with dysuria or ischuria. Before its development there might have been a slight narrowness of the prepuce, or to use a vulgar expression, the patient could only unsheath the penis with difficulty, the opening of the prepuce being very narrow.

Temporary phimosis, as its name indicates, happens in persons who, hitherto, found no difficulty in uncovering the penis, and yields without the necessity of an operation.

Permanent phimosis with excess in the length of the prepuce, or with induration of the circumference of that cutaneous envelope, demands circumcision, unless it is desired to remedy one deformity by creating another. When there are recent adhesions, easy to destroy, it is necessary to dissect them; when they are too intimate and especially too much extended, the prepuce must be removed so as to free the meatus urinarius; when there is excess in the length of the fræenum, the excision of it should be practised; if there are vegetations, they should be removed; if there are chancres, it is better, unless the indications are pressing, that they be cured before the operation, so that the danger of augmenting their extent by inoculating the wound resulting from the operation may be avoided. If the chancres still exist when the operation is practised, they should, as far as practicable, be included in the section. In this way the disease, which can only be local, may sometimes be entirely removed. If, however, some of the chancres still remain, they should be cauterized. When there are perforations of the prepuce, they should be included in the operation.

When the prepuce is short, the section of its superior part by the old method, may suffice. If the prepuce is only narrowed because of vegetations developed between it and the gland, a small incision will answer; in the contrary case, the incision should be carried to a level with the base of the gland. It is necessary to know, however, that even by the superior section, in making the excision of the angles, some patients are left with a strip of lengthened skin, corresponding to the fræenum and constituting a true deformity.

In certain cases, I pinch up longitudinally a fold of the superior part of the prepuce, and remove thus a flap which leaves a division in the form of the letter V, the base of which corresponds to the verge of the prepuce, and the apex to the base of the gland.

As it regards the section of the inferior part of the prepuce, according to the method of Cecus, an operation that M. le Professeur Cloquet has revived and re-created by perfecting it, it is no more liable to wound the urethra, than the superior section. However, I reject it in most cases, especially in phimosis with excess in the length of the prepuce, for it gives place to a deformity in every respect similar to that observed in certain hypospadiœvi.

Giving then the preference to circumcision, this is the method that I employ.

*First.* The penis being in a state of relaxation, without drawing out the skin which forms the prepuce, I trace with ink, a line which follows in its entire circumference, the oblique direction of the base of the gland, and about two lines in advance of that base.

*Second.* This being done, I draw the prepuce forward and fix it between the clamps of the dressing forceps, placed immemediately before the gland and behind the line traced by the ink, the direction of which they follow. The forceps are held by an assistant, the rings being on the side of the dorsal face of the penis, and not transversely as it has been advised in another method.

*Third.* The portion of the prepuce that protrudes beyond the clamps of the forceps is then seized with the fingers of the left hand of the operator, whilst the right armed with a straight bistoury, makes the section, following the oblique direction of the forceps, which placed before the gland, protects it and serves as a sort of guide to the bistoury.

*Fourth.* After this section, the mucous lining, which on account of its anatomical disposition, cannot be drawn forward like the the skin, remains entire upon the gland which it covers, and should be divided in order to avoid a secondary phimosis or paraphimosis.

In order to perform this part of the operation, I divide, at a single stroke with the scissors, this mucous lining upon the dorsal face of the gland and as far as its base; afterwards seizing, one after another, the flaps resulting from the division that I have just indicated, I practise the excision of each side, following the corona glandis as far as the fraenum; then at a single stroke, holding the two flaps united, I cut the fraenum, which I take away with them.

The consequences of circumcision according to my method, appear to me to be more favorable, than those resulting from any others; of this one may become convinced at the venerial hospital. The cure is usually accomplished in about twenty to twenty-five days; no deformity nor even a consecutive phimosis or paraphimosis ever resulting.

After the operation the artery of the frænum or some of the prepucial branches often bleed freely and should be twisted or ligatured. It is necessary afterward to keep the penis constantly covered with cold water, in order to avoid erections and inflammation. With the view also of preventing erections, pills of camphor are given to the patient.

The uninterrupted suture as a means of union offers no very great advantages.

### OF PARAPHIMOSIS.

Paraphimosis, which is nothing else than phimosis carried behind the gland which it compresses, producing all the accidents that may result from strangulation, requires that the parts be replaced in the normal relations.

When the constriction is not very considerable, a methodical compression will suffice for the reduction; in order to accomplish this, the penis should be enveloped in a compress saturated with cold water; it is then to be taken fully into the hands, and after sufficiently compressing it, the compression should be removed; then taking the penis into the left hand, the base of the gland is to be pressed by the fingers of the right, in order to make it enter the ring behind it which is formed by the prepuce. Another method consists in seizing the penis behind the constriction, between the indicatus and the medius of the two hands, whilst the thumbs compressing the sides of the gland, attempt to force the strangulated tissues over.

When there is œdema, it is necessary to practise punctures, thus disgorging the tissues, before attempting the reduction.

But whenever the strangulation is considerable, or when there are ulcerations of the constricted tissues, or adhesions, or inflammation of the gland, threatning, if not already producing gangrene, and further, when the paraphimosis has succeeded a phimosis;—to follow the common method of reducing it will serve only, amid the most poignant suffering excited by the bad management, to aggravate the difficulties already present, and in all cases to convert a paraphimosis into phimosis, which will afterwards require an operation.

In place of those multiplied incisions that have been advised, I make one upon the dorsal face of the penis which completely divides the skin, beginning at the point of the strangulation, and carrying it backward in length equal to that of the gland. This incision is made with a straight and narrow-bladed bistoury, which is glided under the skin, passing under the bridle which is formed behind the gland by the verge of the prepuce. In relation to the mucous lining of the prepuce, which produces in front of the constriction, an œdematous enlargement more or less firm, it should be completely divided in the same direction.

If I have been understood, the operation that I practice thus, is only the operation for phimosis by the superior part, carried behind the gland; consequently the consecutive cures are those of this last operation.

